# Mugilid Fish Are Sentinels of Exposure to Endocrine Disrupting Compounds in Coastal and Estuarine Environments

**DOI:** 10.3390/md12094756

**Published:** 2014-09-12

**Authors:** Maren Ortiz-Zarragoitia, Cristina Bizarro, Iratxe Rojo-Bartolomé, Oihane Diaz de Cerio, Miren P. Cajaraville, Ibon Cancio

**Affiliations:** Research Centre for Experimental Marine Biology and Biotechnology, Plentzia Marine Station (PIE-UPV/EHU) and Department of Zoology and Animal Cell Biology, Faculty of Science and Technology, University of the Basque Country (UPV/EHU), E-48080 Bilbao PO Box 644, Basque Country, Spain; E-Mails: maren.ortiz@ehu.es (M.O.-Z.); cristina.bizarro@ehu.es (C.B.); iratxe.rojo@ehu.es (I.R.-B.); oihane.diazdecerio@ehu.es (O.D.C.); mirenp.cajaraville@ehu.es (M.P.C.)

**Keywords:** mullets, endocrine disrupting chemicals, xenoestrogenicity, intersex, molecular markers, 5S rRNA, TFIIIA, environmental monitoring

## Abstract

Effects on fish reproduction can result from a variety of toxicity mechanisms first operating at the molecular level. Notably, the presence in the environment of some compounds termed endocrine disrupting chemicals (EDCs) can cause adverse effects on reproduction by interfering with the endocrine system. In some cases, exposure to EDCs leads to the animal feminization and male fish may develop oocytes in testis (intersex condition). Mugilid fish are well suited sentinel organisms to study the effects of reproductive EDCs in the monitoring of estuarine/marine environments. Up-regulation of aromatases and vitellogenins in males and juveniles and the presence of intersex individuals have been described in a wide array of mullet species worldwide. There is a need to develop new molecular markers to identify early feminization responses and intersex condition in fish populations, studying mechanisms that regulate gonad differentiation under exposure to xenoestrogens. Interestingly, an electrophoresis of gonad RNA, shows a strong expression of 5S rRNA in oocytes, indicating the potential of 5S rRNA and its regulating proteins to become useful molecular makers of oocyte presence in testis. Therefore, the use of these oocyte markers to sex and identify intersex mullets could constitute powerful molecular biomarkers to assess xenoestrogenicity in field conditions.

## 1. Introduction

Complex contaminant cocktails arrive at estuarine and coastal areas affecting the health of the organisms inhabiting these special ecosystems. Effects are not exclusive to littoral areas, organisms from offshore and deep sea zones have also been impacted by anthropogenic pollutants [1,2]. Among contaminant families detected in the marine environment, the presence of endocrine disrupting chemicals (EDCs) has received special attention [3,4]. EDCs have been defined as exogenous substances or mixtures that alter functions of the endocrine system and consequently adversely affect the health of exposed organisms, (sub)populations, or their progeny [4,5,6,7].

Contaminants classified as reproductive EDCs include plasticizers, pesticides, fungicides, surfactants, dioxins, polychlorinated biphenyls, electrical transformers, and pharmaceuticals such as the synthetic estrogen 17α-ethinylestradiol [8]. They can also be produced as breakdown products of other chemicals and some are natural occurring compounds, synthesized for instance by plants and fungi such as phyto-estrogens and myco-estrogens [9]. The effects of EDCs in aquatic organisms are well known, feminization of male and juvenile fish being one of the best described effects [4,6,9,10]. Male fish with testis containing oocytes (known as intersex condition) have been described in aquatic environments receiving feminizing EDCs or xenoestrogens. Intersex condition has been defined as the presence of oocytes, individually or in clusters, within testicular tissue [6,11,12,13]. In the most severe cases, completely feminized gonads have been described [11]. Fish individuals showing intersex condition have lower reproduction capacity than non-impacted ones, indicating a threat for population viability [13,14,15]. In extreme and long-lasting xenoestrogen exposure scenarios, collapse of fish populations has been described [16]. Nevertheless, a recent study in roach, *Rutilus rutilus*, populations from UK rivers, did not show any direct association between intersex condition and population dynamics [17]. 

Xenoestrogens can alter normal sexual differentiation and gametogenesis because they can interfere with synthesis, storage, release, transport, metabolism, binding action and/or elimination of endogenous hormones [4,9,18]. Levels of vitellogenin, the precursor molecule of egg-yolk proteins, have been extensively measured as biomarker of xenoestrogenicity in adult male and immature fish [3,9,19]. Vitellogenin is a phosphoglycolipoprotein that once synthesized in the liver is secreted into the blood and transported to the ovary, where it is sequestered to form yolk proteins in growing oocytes [10,20]. Genetically determined sex has only been demonstrated in a few fish species, but even in these species, steroid hormones, either exogenous or endogenous, play a crucial role in sexual differentiation [21,22]. Aromatases, belonging to the P450 cytochrome family of proteins, are responsible for the transformation of androgens into estrogens. They are key proteins in the control of steroid balance during sexual differentiation, development and reproduction [21,23]. Two different aromatase isoforms, encoded by two different genes, have been described in fish. They show distinct regulation mechanisms and tissue distribution; *cyp19a1a* (ovarian aromatase) and *cyp19a1b* (brain aromatase), both synthesizing estrogens from androgens [24,25,26]. Exposure to EDCs can modulate the activity of both aromatases and alter the transcription in target tissues [23,25,27,28]. 

Although in terms of number of species, effects of reproductive EDCs have been mainly reported in freshwater fish [11], marine organisms are also exposed to them. One of the pioneering and more complete surveys of xenoestrogenicity to marine fishes was performed in the 1990s in UK estuaries. Male flounder (*Platichthys flesus*) from industrialized estuaries showed elevated plasma levels of vitellogenin and intersex condition [29,30]. The authors demonstrated that feminized flounders were exposed to effluents from sewage treatment plants containing synthetic estrogenic pharmaceuticals (ethinylestradiol and diethylstilbestrol) and alkylphenols. Some male flounders with elevated vitellogenin concentrations were captured in the open sea, but these were hypothesized to be fish that had recently emigrated from a contaminated estuary [31]. Similar surveys were performed in estuarine and coastal areas of the Netherlands [32,33], USA [34,35], Japan [36,37] and recently in the South Bay of Biscay [38] demonstrating the worldwide extension of xenoestrogenic effects into the marine environment. The effects, however, are not restricted to estuarine and coastal areas, and several studies have reported feminizing effects offshore. Estrogenic effects have been observed in swordfish (*Xiphias gladius*) from the Mediterranean [39] and off the coast of South Africa [40], in tuna (*Thunnus thynnus*), little tuna (*Euthynnus alletteratus*) and red mullet (*Mullus barbatus*) from the Mediterranean Sea [41,42,43], and in elder male cod (*Gadus morhua*) and dab (*Limanda limanda*) from the Northeast Atlantic [44,45]. Teleost fish have been widely used as pollution sentinel organisms [46]. The aim of the present review is to provide an overview describing the potential of the family of mugilids as pollution sentinels. This family of teleosts is very well suited for the study of xenoestrogenic endocrine disruption in coastal ecosystems. We further want to review the functional significance of some novel, 5S rRNA related, molecular markers of intersex condition identified in thicklip grey mullets, *Chelon labrosus,* from polluted estuaries in the Basque coast. 

## 2. Mugilids as Sentinel Species of EDC Pollution 

**Figure 1:**
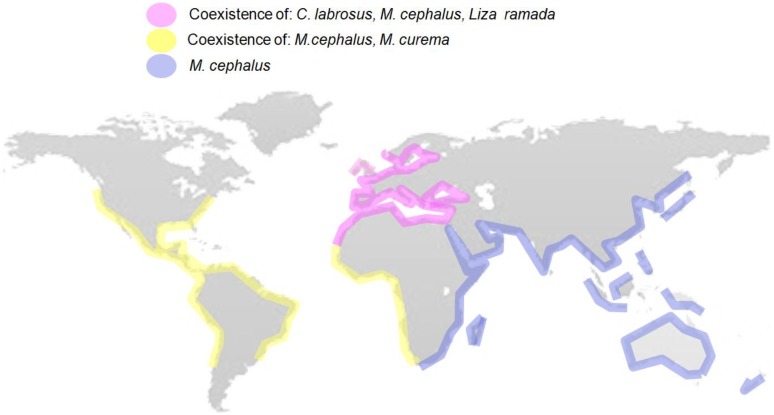
World distribution map of different mugilid fish species showing their association to coastal waters and their wide distribution with the exception of (sub)-Arctic and (sub)-Antartic waters. The most widely distributed species is *Mugil cephalus*. The map illustrates the coexistence of different mullet species in many locations. Out of the four species shown, intersex condition has been described in *M. cephalus. Chelon labrosus* and *Liza ramada*. The map has been produced using the information available in Fishbase [60].

The main characteristics established by Suter in 1993 [47] for a good sentinel species include: widespread distribution, high trophic status, ability to bio-accumulate pollutants, and easy to capture and maintain/study in captivity. It is advisable that they display a restricted home range, with a well-known biology and showing sensitivity but resilience to pollutant exposure. Mugilids are able to endure highly polluted environments, displaying several of the characteristics required for estuarine sentinel species [48,49,50,51]. Their worldwide distribution (Figure 1) and their similar life histories allow the comparison of responses in different geographical areas.

Mullets have been utilized in parasitological [52,53], phylogenic [54] and ecological studies [55] and in environmental health assessment [38,48,56,57,58]. Ferreira *et al*. [56] showed that the presence of pollutants induced oxidative stress responses in *Mugil cephalus* collected in the Douro estuary (Portugal). These mullets showed elevated levels of organochlorine compounds in their tissues [56]. Similar results were obtained in populations analyzed in toxaphene polluted environments in the USA [59]. Alterations of antioxidant enzyme activity levels and peroxisome proliferation were described in mullets collected in the polluted environment of the Arriluze marina located in the mouth of Bilbao estuary, South East Bay of Biscay [58]. All these studies and the UNEP-MEDPOL program, for the assessment of the health of the Mediterranean Sea, recommend the use of mugilids in biomonitoring studies as alternative to other species more difficult to capture [49].

The family Mugilidae is formed by 20 genera, with 80 species, that are distributed worldwide (Supplementary Table S1), principally in seas of mild and tropical climates (Figure 1) [61,62]. All mullet species are pelagic and inhabit numerous habitats including coastal areas, estuaries, rivers, costal lagoons and seas. Mullets are considered gonochoristic but nonfunctional hermaphroditic characteristics have been associated to differentiated mature gonads in *Mugil cephalus* inhabiting coastal waters in South Carolina (USA) [63]. Sex differentiation, in *M. cephalus* and other mullets, begins after 12 months of age and the majority of immature fish at 15–17 months are differentiated sexually [63]. No chromosomal sexual determination system has been established, while environmental and polygenic factors have been suggested to play crucial roles in their sexual determination [21,64]. The sex ratio is 1:1 in most wild populations, but as the population ages a higher prevalence of females is found, probably due to a higher mortality of males [65]. Mullets are considered isochronal spawners; with synchronous gamete development. Individuals spawn all their gametes at once or in batches over a very short period of time [66,67,68].

Mugilids spawn in different periods of the year depending on the latitude and the species, but in all cases reproduction takes place offshore at sea [62,69,70]. After hatching, fry (young-of-the-year) are recruited into monospecific schools in protected coastal areas or in estuaries [70] and later return to littoral waters at ages that range, in the Mediterranean Sea, from 2 months in *Chelon labrosus* to 7 months in *Liza ramada* [69,71], at least. Unlike adults, all the Mugilidae larvae and post-larvae feed mostly on zooplankton; during the recruitment phase both larval (planktivorous) and adult (grazing/detritivorous) feeding strategies coexist in relative proportions, changing according to the food type available [71].

Due to its high ecological plasticity, Mugilidae is one of the dominant fish families in the ecosystems they inhabit [72,73,74,75]. The majority of Mugilidae species are highly euryhaline [76,77] and, being omnivorous, they are able to feed on a great variety of materials including detritus, unicellular algae, crustaceans, mollusks and insects [76,78,79]. This characteristic makes it an ecologically important family for its decisive contribution to the energy and matter flow from the lower to the upper levels in the ecosystems they inhabit [78,79,80]. Several different mugilid species can inhabit the same estuary (Figure 1), as they are able to utilize the food distributed from the thin water surface film to the bottom mud, either by direct grazing or using plant-detritus food chains as an energy source [71]. In fact, salinity gradients along coastal and estuarine environments also allow a zonal distribution of mugilids in a given system [81]. A day and night turnover of different mugilid species in estuaries has also been reported, supporting the co-habitation of several species in the same environment [82].

Mugilids have been widely farmed in aquaculture facilities, with selected target species depending on the region. The commercial importance of mugilids depends on the country and whether they are cultured for gathering roe or for food consumption [71,83]. The total world production of mullet species (fishery and aquaculture) is important and an increasing trend has been evidenced since 1950 [84].

Due to their benthic feeding strategy mullets tend to accumulate more contaminants than other fish species [50,85,86]. Thus, they are ideal organisms for the identification of the levels of metals [49,50,87,88] or organic contaminants such as PCBs and organochlorine compounds [56,85,89] accumulated in tissues. Biological endpoints analyzed in mullets include pollution mediated skeletal deformities [90], oxidative stress and biotransformation enzyme responses [48,91], genotoxic responses [92,93] and endocrine disruption effects (see below). Information about key xenoestrogenicity biomarkers, such as vitellogenin and cyp19a1b aromatase is available for several mullet species. Specific antibody based ELISA tests have been developed for *Mugil cephalus* [94,95], *Liza aurata* [96] and *Chelon labrosus* [38] vitellogenin. The baseline level of plasma vitellogenin concentration in male *M. cephalus* from Korean and Japanese coastal waters has been established at 1 μg/mL [94]. Three different vitellogenins have been identified in *M. cephalus*, vitellogenins A, B and C which, as in other marine teleosts spawning pelagic eggs, are proteolytically-cleaved to different extents as maturation proceeds [97].

Although mullets can endure highly polluted environments, several works indicate that they may be more sensitive to EDC exposure than other fish species. Ferreira *et al*. [48] compared grey mullet (*Mugil cephalus*) and flounder (*Plathychis flesus*) inhabiting the Douro estuary (Portugal) and demonstrated that EROD activity levels in liver were 10 fold higher in mullets than in flounders. Furthermore, in the same study no intersex flounder was described but 21% of studied male mullets displayed intersex gonads. Juvenile so-iuy mullets (*Mugil soiuy*) injected with 17β-estradiol (E2) were more sensitive to vitellogenin mRNA up-regulation than juvenile trout [98]. This higher sensitivity to xenoestrogens was recently corroborated *in vitro* at the molecular level, showing that so-iuy mullet estrogen receptor bound estrogenic hormones estradiol, estrone and estriol with higher affinity than medaka estrogen receptor [99].

**Table 1:** Summary of reports on endocrine disruption in wild populations of mullets. For a more comprehensive description of mentioned endpoints see the text (VTG = vitellogenin).

Species	Common Name	Location	Xenoestrogenicity Endpoints Measured	References
*Chelon haematocheilus*	Redlip mullet	East coast of China (East China Sea)	Intersex condition; VTG protein levels	[100]
*Chelon labrosus*	Thicklip grey mullet	Basque coast (Bay of Biscay)	Intersex condition; VTG protein and mRNA levels; cyp19a1a and cyp19a1b aromatases mRNA levels; oocyte molecular markers; chemical metabolite levels in bile.	[38,101,102]
*Liza ramada*	Thinlip grey mullet	Homa Lagoon (Izmir Bay-Aegean Sea)	Hermaphrodite (intersex) gonads	[103]
*Mugil cephalus*	Flathead grey mullet	Douro estuary (Portugal, East Atlantic coast)	Intersex condition	[48,56]
		Orbetello Lagoon-West Italy (Mediterranean Sea)	VTG mRNA levels; oocyte development and atresia	[104,105,106]
		South coast of Korea (East China Sea)	Intersex condition; VTG protein levels	[94,100]
		South coast of Japan (East China Sea)	Intersex condition; VTG protein levels	[94,100]
		East coast of China (East China Sea)	Intersex condition; VTG protein levels	[100]
*Mugil soiuy*	So-iuy mullet/Far Eastern mullet	North East coast of China (Bo Sea)	VTG mRNA levels	[98]

## 3. Effects Mediated by EDCs in Mugilids

The presence of ovotestis in mullets was described for the first time in the Ligurian Sea a century ago [107], but no potential causes were proposed. This fish, identified as *Mugil chelo* currently accepted name *Chelon labrosus*, contained ovarian lobes and testicular areas in the same gonad. Recent studies have reported the presence of intersex mullets in polluted estuaries worldwide (Table 1). Thus, intersex *Liza ramada* was described in waters polluted with industrial and agricultural outfalls in Turkey [103]. Intersex *Mugil cephalus* were found in estuaries from Korea, Japan, China and Portugal [48,94,100]. The testis of these fish showed dispersed previtellogenic or vitellogenic oocytes, suggesting exposure to xenoestrogenic compounds. Elevated vitellogenin levels, measured as plasma protein levels or liver mRNA transcript levels, were detected in male mullets from the same places where the intersex mullets had been collected. Similarly, in *Mugil soiuy* from Bo Sea (North China), polluted with urban and industrial effluent discharges, hepatic up-regulation of vitellogenin transcription was described [98].

**Figure 2:**
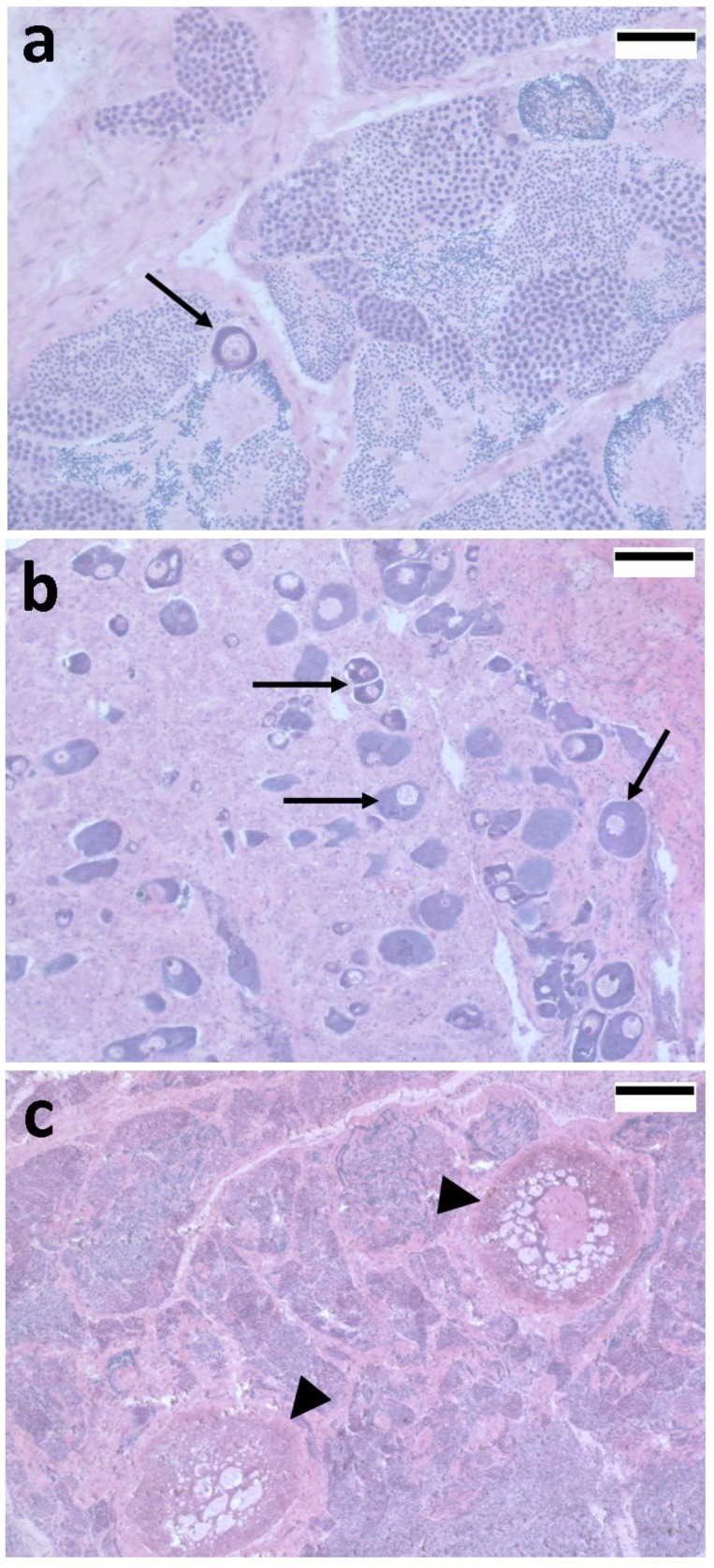
Photomicrographs showing hematoxylin-eosin stained histological sections of thicklip grey mullet (*Chelon labrosus*) intersex gonads from South East Bay of Biscay; (**a**) Single previtellogenic oocyte (arrow) within testicular tissue at advanced gametogenic stage; (**b**) Presence of multiple previtellogenic oocytes (arrows) within testicular tissue at early gametogenic stage; (**c**) Vitellogenic oocytes (arrowheads) within testicular tissue at mature stage. Scale bars are 50 µm (**a**), 100 µm (**b**) and 200 µm (**c**).

Intersex gonads have also been described in *Chelon labrosus* (Figure 2) from estuaries in the South Bay of Biscay [38,101,102]. Oocytes have been found in male mullets from Bilbao, Pasaia and Ondarroa harbors, from the Deba river estuary and from the Biosphere Reserve of the Urdaibai estuary in Gernika [38,101]. Intersex mullets were found all along a reproductive annual cycle, with prevalences ranging from 3% to 60% of analyzed male mullets at each month [108]. Most *C. labrosus* showing intersex condition contained dispersed or small clusters of previtellogenic oocytes in testes (Figure 2) and were classified as presenting low to moderate intersex severity according to the index developed by Jobling *et al*. [109]. Elevated vitellogenin plasma and transcription levels were detected in these *C. labrosus* populations, accompanied by up-regulation of *cyp19a1b* transcription levels in brain [101]. Furthermore, the detection of high levels of alkylphenols and synthetic estrogenic hormones, such as ethinylestradiol, in mullet bile demonstrated the exposure of these populations to xenoestrogenic compounds [38,101]. 

No statistical correlation was found between intersex condition and vitellogenin levels in mullet species [95,101]. Bahamonde *et al*. [11] suggested that additional mechanisms not directly estrogen dependent, should be involved in the development of intersex condition in fish. Other steroidogenic and neuroendocrine dependent pathways could be involved in the first steps of intersex gonad development. However, it is known that during puberty *cyp19a1b* expression in brain and *cyp19a1a* in ovary increases in mullets, promoting female sexual differentiation in *M. cephalus* [110]. Similarly, Bizarro *et al*. [101] found that *C. labrosus* showing intersex condition had higher *cyp19a1b* transcription levels in brain than non-intersex males from the same population. Further work will help to elucidate the role played by aromatases and other steroidogenic genes and enzymes in the development of intersex condition in fish.

Anti-estrogenic effects have also been described in wild populations of mugilids. *M. cephalus* from the Orbetello Lagoon (West Italy), showing low brain acetylcholinesterase activity due to exposure to organophosphate and carbamate compounds, presented ovaries with underdeveloped and deformed oocytes [104]. Accordingly, no vitellogenin transcription was detected in the liver of these mullets [105,106].

Mugilids are also suitable as experimental coastal fish to assess the action mechanisms and the effects of EDCs (Table 2). Several laboratory studies have focused on the effects of steroid hormones in the gametogenesis and sexual differentiation of mullets. Onset of oogenesis and induction of gonad aromatase activity have been demonstrated in juvenile *M. cephalus* after exposure to E2 during sexual differentiation [111]. Aoki *et al*. [112] accelerated oocyte differentiation and obtained complete feminization by feeding juvenile *M. cephalus* diets containing 4 μg/g ethinylestradiol (EE2). These hormonally treated mullets showed a parallel increase in plasma vitellogenin levels that was sustained even after having been placed in clean water for 149 days. Induction of aromatase activity in the gonads and complete feminization were also described in six month old juvenile mullets exposed to EE2 for four months [113]. These fish showed elevated plasma testosterone levels but no changes on estradiol levels were reported, although reduced levels of 11-ketotestosterone, the main active androgen in fish, were quantified after EE2 exposure [113]. On the other hand, exposure to 17α-methyltestosterone induced complete masculinization, 11-ketotestosterone and testosterone plasma levels remaining high, while the majority of control animals at the age of 10 months, when the exposure experiment finished, remained undifferentiated [113]. Similarly, long-term exposure of juvenile *M. cephalus* to methyltestosterone through food, caused complete sex reversal when exposure took place 6–9 month after fertilization, the critical time for sexual differentiation [114]. Nevertheless, at sexual maturity (three years) some adult mullets were able to reverse the hormonally induced sex reversal [114]. Thus, exposure to EDCs affects sexual differentiation, and possibly intersex condition, depending on the time of exposure during mullet development. There is a sensitivity window along larval development, in which a transitory exposure to a low concentration of estrogenic EDCs can feminize male fish but after sex differentiation, the plasticity of fish gonads decreases. This window typically occurs during the first few months of larval development [21], a period spent offshore by mullet species. The late onset of sexual determination/differentiation in mugilids [21] thus, can coincide with the recruitment of fry (2–7 months, depending on the species) into estuarine waters, where they would be exposed to the large concentrations of chemicals typical of these highly anthropogenized environments.

**Table 2:** Mullet exposure to endocrine disrupting chemicals under laboratory conditions. For a more comprehensive description of mentioned endpoints and responses see the text ( GSI = gonado-somatic index; VTG = vitellogenin).

Species	Common Name	Treatment	Effect Endpoints Measured	References
*Chelon* *haematocheilus*	Redlip mullet	Nonylphenol (0.01, 0.1, 1, 10 & 100 ng/mL)	Steroid levels in cultured oocytes	[115]
		PCB126 (0.01, 0.1, 1, 10 & 100 ng/mL)		
*Chelon labrosus*	Thicklip grey mullet	Perfluorooctane sulfonate (2 mg/L)	VTG and cyp19a1b aromatase mRNA	[116]
		Heavy fuel oil (150 mL/11 kg sediment)	levels	
*Liza aurata*	Golden grey mullet	17β-estradiol (2 μg/L & 0.07 mg/kg body weight)	VTG mRNA levels	[117]
		Nonylphenol (25, 100, 1000 μg/L & 0.25, 250 mg/kg body weight)		
*Mugil cephalus*	Flathead grey mullet	17β-estradiol (1, 8, 15 & 120 mg/kg feed)	Oocyte development; GSI	[111]
		17α-ethinylestradiol (20 mg/kg feed)	Gonad development; GSI; steroid plasma levels; gonad aromatase activity.	[113]
		17α-ethinylestradiol (0.04 & 4 μg/kg body weight)	Gonad development; VTG protein levels.	[112]
		17α-methyltestosterone (20 mg/kg feed)	Gonad development; GSI; steroid plasma levels; gonad aromatase activity.	[113]
		17α-methyltestosterone (5, 10 & 15 mg/kg feed)	Gonad development; VTG protein levels	[114]
		17α-methyltestosterone (4 mg/kg body weight)	Gonad development, steroid plasma levels,	[118]
		Domperidone (5 mg/kg body weight)	VTG protein levels	
		GnRH (10 μg/kg body weight)		
		Domperidone (5 mg/kg body weight) + GnRH (10 μg/kg)		

Further to this and at the molecular level, it has been reported that the responses of mullets to EDCs are developmental and gametogenic stage specific. In juvenile *L. aurata*, waterborne exposure to E2 (2 μg/L) for one week did induce vitellogenin production, although hepatic down-regulation of *cyp1a1* and inhibition of EROD activity were observed. However, adults intraperitoneally injected with 0.07 mg/Kg E2 showed elevated plasma levels and up-regulation of vitellogenin. Similar life history dependent opposite results were obtained when juvenile and adult *L. aurata* were exposed to nonylphenol [117]. In another study, exposure of *Chelon haematocheilus* vitellogenic oocytes *in vitro* to nonylphenol inhibited estradiol synthesis and stimulated testosterone production [115]. The authors suggested a potential anti-estrogenic effect of nonylphenol on mature oocytes (0.75 μm), since exposure of pre-mature oocytes (0.65–0.75 μm) did not elicit such response. No effects of PCB126 were detected in the same experimental model [117]. Oocyte development not only relies on estrogen levels, other neuroendocrine factors play an important role in the regulation of oogenesis. Aizen *et al*. [118] demonstrated that by inhibiting dopamine dependent response in adult *M. cephalus*, oocyte development and maturation were arrested. This suggests that mullet populations inhabiting waters polluted with dopamine antagonist drugs, such as domperidone, can show altered reproductive and gametogenic cycle. Transient responses to contaminants have been described in mullets that regulate their adaptive mechanisms and pathways which, in turn, might allow their survival in polluted environments. Juvenile *C. labrosus* exposed to perfluorooctanesulfonic acid and heavy fuel oil showed no changes of vitellogenin transcription levels but up-regulation of *cyp19a1b* and down-regulation of estrogen receptor alpha in the brain after two days of exposure were observed [116]. However, after 16 days of exposure no significant changes in the transcript levels of these genes were detected. 

## 4. Novel Intersex Markers in Mullets

Sex determination results in the beginning of the differentiation process allowing bipotential primordial germ cells (PGCs) to produce either oocytes or sperm in the maturing fish. Female teleosts maintain germline stem cells for life, so they can produce an unlimited number of eggs during their reproductive lifetimes [119,120,121]. Timing of oocyte differentiation in females is controlled by estradiol and 11-ketotestosterone among other hormones. In each reproductive cycle PGCs transform into oogonia that initiate proliferation. When entering meiosis these oogonia render multiple primary oocytes, arrested in meiotic prophase [122]. Differentiation continues under hormonal control and as the oocyte grows, it accumulates cortical alveoli. These previtellogenic stage oocytes are also termed perinucleolar stage oocytes due to the proliferation of perinucleolar structures, structural manifestation of ribosomal RNA production. The development of the oocytes may be arrested at this stage until they are recruited for secondary growth, which results in massive growth during vitellogenesis, whereby the oocyte accumulates nutritional reserves, completing the differentiation of its cellular and non-cellular envelopes. If the oocyte recruitment stops before the spawning (mature egg) season then the species is considered to be a determinate fecundity species (synchronous, as in mugilids, gadoids, pleuronectoids or clupeids), while the recruitment continues until the postspawning period in the indeterminate fecundity species (anchovies, hakes, mackerels). In one case all oocytes display the same stage in the ovary (total spawners) as in mugilids, while in the others, oocytes in different maturation stages coexist in the ovaries (batch spawners) [123]. 

The term vitellogenesis describes the incorporation of hepatic vitellogenins into oocytes and their processing into yolk proteins [124]. Vitellogenin and egg envelope proteins are obviously oocyte markers, but only during vitellogenesis and not during the long period of the year in which oocytes remain in previtellogenic stage. This is also true for oocytes in intersex individuals, usually found in perinucleolar stage. Differentiation encompasses the whole process that allows the oocyte to become competent to undergo fertilization, through incorporation of maternal RNAs, proteins, lipids, carbohydrates, vitamins and hormones that are important for the proper development of the embryo. Each one of such molecules constitutes a marker of oocyte differentiation and in theory should constitute a marker of intersex condition in male fish producing oocytes in their testis. The high prevalence of intersex males in mullet populations inhabiting polluted estuaries in the Basque Country has provided opportunities of discovering such molecular markers of oocyte development in testis [101,102,108], which we need to understand functionally.

### 4.1. 5S rRNA as a Sex Marker in Fish Gonads: A Crucial Molecule in Oocyte Differentiation

**Figure 3:**
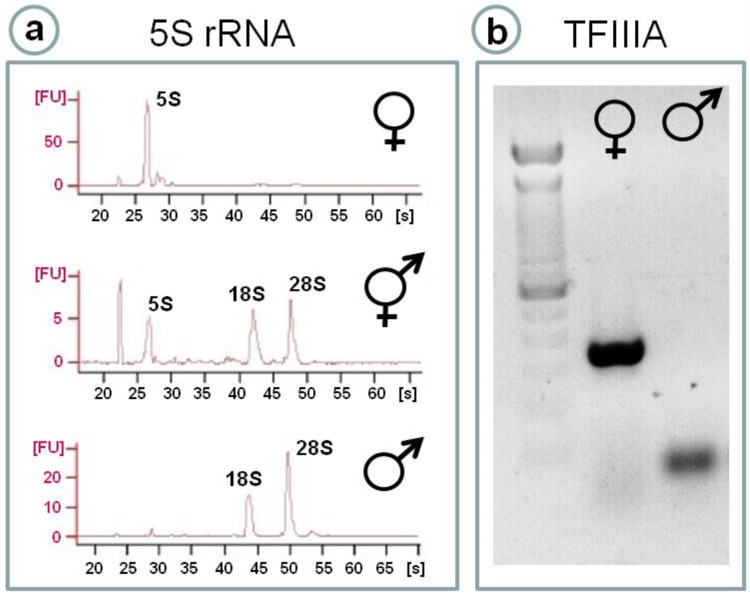
(**a**) Typical electropherograms obtained from the electrophoretic analysis (Bioanalyzer 2000, Agilent Tech., Santa Clara, CA, USA) of total RNA extracted from the gonads of a female, a male and an intersex male thicklip grey mullets (*C. labrosus*). The 5S rRNA peak is clearly observed in ovary and in intersex testis, while the eukaryotic typical 18S and 28S rRNA peaks can be observed in testis; both normal and intersex. Note that the ovary shown belongs to a female in previtellogenic stage; (**b**) DNA electrophoresis showing the Q-PCR product obtained after specific amplification of TFIIIA cDNA from *C. labrosus* ovary (present) and testis (not present). The ghost band in testis is the result of primer dimmers.

A simple electrophoresis of total RNA extracted from gonads of thicklip grey mullets *C. labrosus*, is enough to identify their sex depending on the apparition or not of a potent low Mw band of 120 bp (Figure 3). This is so, irrespective of the moment along the reproductive cycle and site (polluted or not) of collection of samples. This band belongs to the smallest ribosomal RNA molecule, 5S rRNA [102]. In particular, 5S rRNA could constitute 75% of the total RNA content in a female gonad. Moreover, and as strong expression of 5S rRNA can be considered a marker of the presence of oocytes, intersex *C. labrosus* individuals from polluted estuaries can be identified due to their levels of 5S rRNA expression in between males and females [102]. It seems that oocytes need to accumulate 5S rRNA in order to quickly assemble ribosomes in case of being fertilized and sustain protein synthesis during embryogenesis.

Similar observations in the RNA profile of fish ovaries had been reported by Mazabraud *et al*. [125] and Denis and Wegnez [126] describing that in four out of nine teleost species studied tRNA and 5S rRNA made up more than 90% of the RNA content of the ovaries. Mittelholzer *et al*. [127] studying the gene transcription profiles of aromatase and 20β-hydroxysteroid dehydrogenase in Atlantic cod ovary, described large peaks, of around 100 nucleotides in size, suggested by authors to belong to 5S rRNA, that completely masked the normally predominant 18S and 28S rRNA peaks. The same description of small RNA molecules appearing on an electrophoresis of roach *Rutilus rutilus* ovarian total RNA was reported by Kroupova *et al*. [128]. In previtellogenic oocytes of teleosts, 5S rRNA is stored together with tRNA in ribonucleoprotein particles (RNPs) of different sizes [125]. 

Early vitellogenesis in amphibian oocytes is characterized by a dramatic increase in the expression of 5S rRNA genes whereas transcription is slowed down in later stages [129]. These variations in rRNA gene activity during oogenesis are accompanied by well-characterized ultrastructural modifications of the amphibian nucleolus. Fish also display a succession of transformations in nucleolar morphology during the differentiation of oogonia into mature oocytes, very similar to those in anurans [130]. The previtellogenic or perinucleolar oocytes are characterized by the richness in nucleoli [124].

The 5S ribosomal RNA gene family is arranged in higher eukaryotes in several copies of tandem repeated units. Each unit consists of a highly conserved coding sequence of 120 bp, and a flanking non-transcribed spacer (NTS) [131,132,133]. This NTS has been employed as molecular marker for species identification [131,132]. Many studies have been published dealing with the structure, chromosomal location, and sequence variation of the 5S rRNA genes in fish, including grey mullets [131,132]. Two different types of 5S rDNA have been found in the frog *Xenopus laevis*, one expressed in somatic cells and the other in oocytes [134]. This system of two paralogous 5S rRNA gene classes is also common to fish species [126,131,135]. This has been assumed to reflect the existence of a gonadal and a somatic gene in fish, although the distinction so far has been restricted to describe the different 5S rDNA tandem repeats as organized in two distinct size-classes [131,135,136,137]. 

### 4.2. Transcriptional Regulation of Ovarian 5S rRNA and Storage of Ribosomal Subunits

Q-PCR analysis of thicklip grey mullet demonstrated, for the first time in a fish species, that the transcriptional regulation of an ortholog of the general transcription factor IIIA (TFIIIA) resembled that of 5S rRNA in gonads. Intersex individuals showed transcription patterns in between both sexes [102], which makes TFIIIA also a potent molecular marker of oocytes. Interestingly, TFIIIA was also reported in a list of ovary-expressed genes in zebrafish [138].

**Figure 4:**
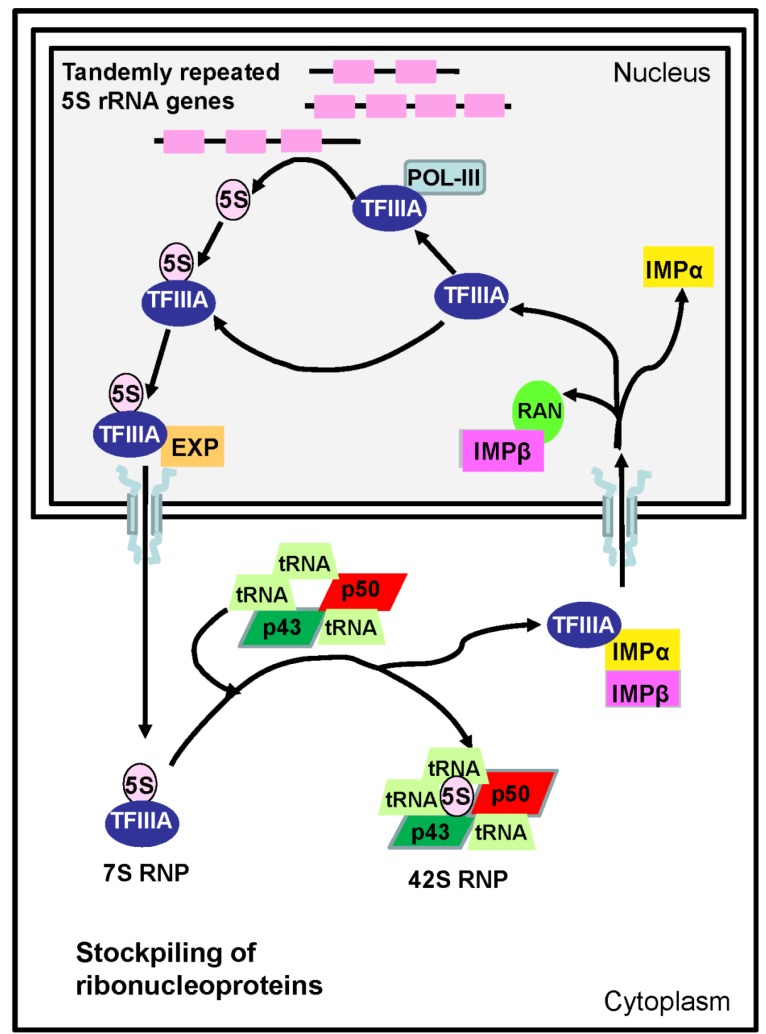
Schematic representation of the processes and molecules involved in the transcription, nucleo-cytoplasmic transport and stockpiling of 5S rRNA in anuran and in fish oocytes (modified from [133]).

TFIIIA controls the transcription of 5S rRNA by RNA polymerase III in eukaryotes [133]. A single TFIIIA gene codes for two different isoforms, of 38 and 40 kDa in *Xenopus* [139]. These isoforms correspond to the oocyte and somatic form of the protein, synthesized through differential promoter usage [129,139]. In *Xenopus* oocytes, TFIIIA forms a complex with 5S rRNA (7S RNP) that serves as a storage particle for 5S rRNA (Figure 4). Thus, TFIIIA specifically recognizes and binds the 5S rDNA promoter sequence, and also binds the resulting transcription product. Levels of TFIIIA mRNA mirror those of total 5S rRNA and, in *Xenopus*, are approximately one million times higher in oocytes than in somatic cells [129]. TFIIIA is overexpressed early in oogenesis (stages I–III), the protein constituting as much as 10% of the total cytoplasmic protein in anurans [129], and then decreases 5–10-fold by stage V–VI [139]. 

In fish, TFIIIA has only been characterized in the catfish, where it was shown to be associated to 5S rRNA in ovarian tissue [140]. Although the 38 kDa protein only displays 40% aa sequence identity with *Xenopus* TFIIIA, it is able to bind *Xenopus* 5S rRNA genes. The possibility that two independent *tfIIIa* paralogous genes, one shorter than the other, exist in fish genomes can be suggested analyzing the fish genomes in ENSMBL (in zebrafish: *gtf3aa* ENSDARG00000030267, chromosome 5, protein size 367 aa and 42.6 kDa and *gtf3ab* ENSDARG00000071583, chromosome 24, protein size 318 aa and 37.15 kDa). This opens the possibility to an ovarian specific TFIIIA gene, and an independent somatic gene, in teleost genomes. 

Similarly to TFIIIA, mullet p43 or 42sp43 was transcribed to very high levels in ovaries of *C. labrosus*, during the entire reproductive cycle of females and in intersex males [102]. In addition to 7S RNP complexes, 5S rRNA is also stored in larger 42S RNPs (Figure 3). In *Xenopus*, approximately 50% of the 5S rRNA in oocytes is associated with TFIIIA, the other half being present in 42S RNPs. These RNPs consist of two proteins: 42sp50 (also termed p48) and 42sp43 (also termed p43 or thesaurin b). 42sp43 is a nine zinc-finger protein responsible for 5S rRNA binding, but unlike TFIIIA it is not able to bind the gene and it is not implicated in its transcriptional regulation [133]. The only reference in fish is about *Oryzias latipes* where both *42sp50* and *42sp43* gene transcripts were found to be ovary specific [141,142]. A transgenic medaka line constructed using a relative short upstream sequence and 3’UTR from the *42sp50* gene allowed oocytes to be fluorescently labeled; fluorescence beginning as early as five days after hatch [142]. Oocytes artificially induced in the testes of medaka were also fluorescently labeled [142]. 

### 4.3. Nucleo-Cytoplasmic Transport of Proteins and Ribonucleoproteins during Oocyte Differentiation

In eukaryotic cells, nucleo-cytoplasmic bidirectional transport of molecules relies on access through the nuclear pore complexes via carrier proteins, karyopherins [143]. They include members of the importin (IMP) and exportin (EXP) protein families. The best characterized mechanism of nuclear import is mediated by IMPα and IMPβ1 heterodimers (Figure 4). Nuclear proteins bind to IMPα, and this complex is targeted by IMPβ1 to the nuclear pore. In this way, hundreds of different proteins can be specifically targeted into the nucleus. The IMPα gene family has expanded during evolution with a single gene in budding yeast, three in *Drosophila melanogaster* and *Caenorhabditis elegans* (*impα1* to *3*) and six IMPα genes in humans [143,144].

Little is known about this transport system in fish. An IMPα, most similar to *Xenopus* IMPα1 and 2 and including an IMPβ-binding domain, was characterized in the red seabream *Pagrus major* ovary [143]. RT-PCR analysis showed transcription of this *P. major* IMPα in testis, and especially in ovary, but not in other tissues. Three importin (*impα1* and *2* and *impβ2*) and four exportin (*exp1*, *5*, *6* and *7*) fragments have been cloned in *C. labrosus* and both *impα1* and *2* followed the pattern of transcription of TFIIIA, constituting also potent sex and intersex (oocyte) markers [102]. 

A systematic analysis of IMPα variants in *Xenopus* (*impα1* to *4*, *impα5.1* and *5.2*) revealed that *impα1* and *2* are transcribed in a pattern similar to *tfIIIa* during early oogenesis [145]. So TFIIIA could be imported into nuclei via interaction with these two proteins. A quick search of the different fish genomes available in ENSEMBL reveals 7 IMPα genes (termed *kpna1* to *7* for karyopherins), but it is not known how these other IMPα genes could be regulated during fish oogenesis and xenoestrogenicity causing intersex.

### 4.4. How Is 5S rRNA Related Gene Expression Regulated in Fish Oocytes? 

Gene expression processes occurring during early oogenesis/spermatogenesis have to deal with DNA packaged under special structural circumstances while it is involved in the rearrangements occurring at meiosis. It has been reported that germ cells use alternate forms of core promoter transcription factors such as TBP, TAFs, and TFIIA [146]. Genes that encode variants of TFIIA, TBP, and TAFs have been discovered in male and female reproductive tissues [146] and have roles in spermatocytes, oocytes, and the early embryo. Some genes switch from a somatic promoter to one or more germ-cell-specific promoters. This is the case of *tfIIIa* gene in *Xenopus* [129] although we might be facing two separate genes in fish. The capacity to drive oocyte/sperm specific activation of *tfIIIa* transcription (under normal developmental and under exposure to EDCs causing intersex condition) and therefore 5S rRNA production seems central for oocyte differentiation in fish. A comparison of the germ cell specific promoters reveals that they all possess a small size (100 bp). This is exemplified by the transgenic *O. latipes* line [142] with fluorescent ovaries produced using the short promoter sequence of *42sp50*. It is not clear how such short promoters are able to specify germ cell activation and somatic silencing but they suggest that enhancer-dependent interactions are not required. 

Certain regulatory factors, including Sp1, USF, NF-κB, CREMτ, BORIS, SREBP2 or FIGα have been proposed to regulate genes in germ cells [146]. Sp1, USF and Vg1-RBP are known to bind the *tfIIIa* promoter region in *Xenopus* [128]. Activation of a *tfIIIa* control element, termed E3, is essential in specific gene transcription in *Xenopus* early oocytes. Gel shift assays have demonstrated that *tfIIIa* expression correlates with an E3 binding protein (termed the B3 activator and identified as Vg1-RBP) activity, with the highest levels in immature oocytes and very little in somatic cells [147]. Consequently Vg1-RBP may be an important stage specific transcriptional regulator of *tfIIIa* [147].

Questions that remain to be answered in mullets and in fish in general are:

- Which are the dynamics of activation of these oocyte markers during normal female sexual determination/differentiation?
- Which are the environmental (EDC exposure among them) and molecular clues, epigenetic mediators and pathways that regulate activation of 5S rRNA related genes so as to mask any other RNA production in PGCs that initiate differentiation into oogonia instead of spermatogonia?
- Could the levels of 5S rRNA accumulated into oocytes give information about the effects of EDCs on oogenesis and oocyte quality?
- Would these molecular markers function as intersex markers in other mugilid fish species, or in fish species with asynchronous gamete development?

## 5. Conclusions

Within the mugilidae family of fish we find worldwide distributed and strategically placed euryhaline species that are very sensitive to exposure to xenoestrogenic compounds, both under natural and laboratory conditions. Although most mullet species reproduce in offshore marine waters, they return to littoral waters, mainly estuarine brackish waters, within 2–7 months after hatching. It would be then, coinciding with the period of juvenile sex differentiation, that they are first exposed to chemical pollutants in the water bodies receiving the chemicals coming from human settlements and activities. In this respect, intersex male individuals have been widely documented in different mullet species. Vitellogenins are the molecular markers traditionally used in monitoring of xenoestrogenic exposure, but they are not produced during the long lasting previtellogenic stages of oogenesis in most fish species. We need molecular markers able to identify mild intersex conditions all along the reproductive cycle. In this respect, and as it has been proved in intersex mullets captured in estuaries with high levels of EDCs, 5S rRNA and related proteins accumulated in all oocyte stages, could be such helpful markers.

## Supplementary Files

Supplementary File 1
