# Supplementary material for: Mugilid Fish Are Sentinels of Exposure to Endocrine Disrupting Compounds in Coastal and Estuarine Environments
Source: Mar Drugs. 2014 Sep 12;12(9):4756–82. doi: 10.3390/md12094756 (PMC4178482; doi:10.3390/md12094756)
Supplement: Supplementary File 1 [file marinedrugs-12-04756-s001.pdf]

Supplementary Information

Table S1. List of *Mugilidae* family taxonomic groups (genus), species and geographical distribution. Data was obtained from Fishbase [1], WoRMS [2], EOL [3] and ITIS [4] online databases.

Genus	Species	Common Name	Distribution
Agonostomus	*A. catalai*	Comoro mullet	West Indian Ocean (Madagascar and Comoro Islands)
	A. monticola	Mountain mullet	West Atlantic and Caribbean Sea (South USA to Colombia, Venezuela and West Indies)
	A. telfairii	Fairy mullet	West Indian Ocean (Madagascar, Reunión, Mauritius, Seychelles, Anjuan and Comoro Islands)
Aldrichetta	*A. forsteri*	Yellow-eye mullet	South-West Pacific (New Zealand, Chatham Islands, Australia and Tasmania)
Cestraeus	*C. goldiei*	Goldie river mullet	South-Asia and Oceania (South-Philippines and New Caledonia)
	C. oxyrhyncus	Sharp-nose river mullet	South-Asia and Oceania (East-Indonesia, Fiji; Philippines and New Caledonia)
	C. plicatilis	Lobed river mullet	South-Asia and Oceania (Celebes, New Caledonia, New Hebrides, and Fiji)
Chaenomugil	*C. proboscideus*	Snouted mullet	Eastern Central Pacific (Revillagigedo Islands and Mexico to Panama)
Chelon	*C. bispinosus*	Cape Verde mullet	Eastern Central Atlantic (Cape Verde)
	C. labrosus	Thicklip grey mullet	Eastern Atlantic (Scandinavia, to Senegal and Cape Verde). Mediterranean Sea, South-West Black Sea
	C. planiceps	Tade gray mullet	Indo-Pacific (Red Sea to Australia)
Crenimugil	*C. crenilabis*	Fringelip mullet	Indo-Pacific (Red Sea and East Africa to Japan and Lord Howe Island)
	C. heterocheilos	Half fringelip mullet	Indo-Pacific (Indonesia and Philippines to Vanuatu, Japanese Islands and New Caledonia
Joturus	*J. pichardi*	Bobo mullet	Central America (Florida, Mexico to Panama and the West Indies)

Table S1. *Cont.*

			South-West Asia (Iran and Pakistan)
Liza	*L. abu*	Abu mullet	North-West Pacific (Japan, Ryukyu Islands, Taiwan, East China)
	L. affinis	Eastern keelback mullet	Indo-West Pacific (Timor Sea, New Guinea and Tonga)
	L. alata	Diamond mullet	Indo-West Pacific (West-South Australia)
	L. argentea	Flat-tail mullet	Eastern Atlantic (Scotland to Cape Verde, Mediterranean Sea and Black Sea)
	L. aurata	Golden grey mullet	Eastern Central Atlantic (Senegal, Gambia and Guinea Bissau)
	L. bandialensis	Dissanga mullet	Indian Ocean (Red Sea to India, Japan and China).
	L. carinata	Keeled mullet	Eastern Atlantic (Mauritania to South Africa), Western Indian Ocean (Mozambique to South Africa)
	L.dumerili	Groove mullet	Eastern Atlantic (Mauritania to Congo and Angola)
	L. falcipinnis	Sicklefin mullet	Eastern Atlantic (Mauritania to Equatorial Guinea)
	L. grandisquamis	Large scaled mullet	North-West Pacific (Japan, Korean Peninsula and North-East China). Introduced in Black Sea
	L. haematocheila	So-iny mullet	Western Indian Ocean (Persian Gulf to India)
	L.klunzingeri	Klunzinger ́s mullet	Indo-Pacific Oceans (East Africa, India, Indonesia, China, Philippines, Japan, Melanesia and Polynesia)
	L. macrolepsis	Largescale mullet	Indian Ocean (India)
	L. mandapamensis	Indian mullet	Indo-Pacific Oceans (East Africa, Philippines, South China Sea, Tonga and tropical Australia)
	L. melinopterus	Otomebora mullet	Indian Ocean (Pakistan, India, Sri Lanka and Andaman Islands)
	L. parsia	Goldspot mullet	Western Indian Ocean (Bahrain and Qatar)
	L. persicus	Persian mullet	Eastern Atlantic Ocean (Southern Norway to Morocco, Mediterranean Sea and Black Sea
	L. ramada	Thinlip grey mullet	Western Central Pacific (Queensland, Australia)
	L. ransayi	Ramsay ́s mullet	South-East Atlantic (South Africa).
	L. richardsonii	South African mullet	Eastern Atlantic (France to Morocco), Mediterranean Sea, Black Sea and Sea of Azov. Introduced
	L. saliens	Leaping mullet	Caspian Sea
	L. subviridis	Greenback mullet	Indo-Pacific Oceans (Red Sea to Samoa, Japan)
	L. tricuspidens	Striped mullet	South-East Atlantic (Mossel Bay and Kosi Estuary in South Africa and Angola)
	L. vaigiensis	Squaretail mullet	Indo-Pacific Oceans (Red Sea, East Africa, Tuamoto Islands, Japan, Great Barrier Reef and New Caledonia)

Table S1. *Cont.*

Moolgarda	*M. pedaraki*	Longfin mullet	Indo-West Pacific (East Africa to South Africa and East to Western Pacific)
	M. perusii	Longfinned mullet	Indo-West Pacific (East Africa to the Mariana Islands)
	M. seheli	Bluespot mullet	Indo-Pacific Oceans (Red Sea to South Africa, Hawaiian and Marquesan islands, Japan and New Caledonia)
Mugil	*M. bananensis*	Banana mullet	Eastern Atlantic (Senegal to Angola)
	M. broussonnetii	Broussennet ́s mullet	Pacific Ocean (Southern China to South Pacific)
	M capurrii	Leaping African mullet	Eastern Central Atlantic (Morocco to Guinea-Bissau and Togo)
	M. cephalus	Flathead grey mullet	Eastern Pacific (California to Chile), Western Pacific (Japan to Australia), Western Indian Ocean (India to South Africa), Western Atlantic (Nova Scotia to Brazil, not in Caribbean Sea), Eastern Atlantic (Bay of Biscay to South Africa), Mediterranean Sea and Black Sea
	M. curema	White mullet	Western Atlantic (Nova Scotia to Argentina), Eastern Atlantic (Senegal to Namibia), Eastern Pacific (California to Chile)
	M. curvidens	Dwarf mullet	Western Atlantic (West Indies to Brazil), South-East Atlantic (Ascension Island)
	M. galapagensis	Galapagos mullet	South-East Pacific (Galapagos Islands)
	M. gyrans	Whirligig mullet	West Atlantic (Gulf of Mexico)
	M. hospes	Hospe mullet	Western Atlantic (Belize to Brazil), Eastern Pacific (Mexico to Ecuador)
	M. incilis	Parassi mullet	Western Atlantic (West Indies and Central America to South-East Brazil)
	M. liza	Lebranche mullet	Western Atlantic (Bermuda, Florida, Bahamas, Caribbean Sea and North Brazil)
	M. platanus	n.d.	South-West Atlantic (South East Brazil, Uruguay and East Argentina)
	M. rammelsbergii	Yellow-tailed mullet	South-East Pacific (Galapagos Islands and Peru)
	M. rubrioculus	Redeye mullet	Western Atlantic (Central America), Caribbean Sea.
	M. setosus	Liseta mullet	Eastern Central Pacific (Mexico and Revillagigedo Islands).
	M. soiuy	Far Eastern mullet	North-West Pacific (Russia, Japan, Korea and China). Introduced to Azov Sea, Black Sea and Mediterranean Sea
	M. trichodon	Fantail mullet	Western Atlantic (Florida to North-East Brazil)
Myxus	*M. capensis*	Freshwater mullet	South-West Indian Ocean (South Africa)
	M. elongatus	Sand grey mullet	South-West Pacific (Australia)
	M. petardi	Pinkeye mullet	South-West Pacific (Australia)

Table S1. *Cont.*

Neomyxus	*N. chaptalii*<br>*N. leuciscus*	Uouoa mullet<br>Acute-jawed mullet	Eastern Central Pacific (Hawaiian Islands)<br>Pacific Ocean (Southern Japan, Mariana and Bonin Islands, Hawaiian Islands, Micronesia and French Polynesia
Oedalechilus	*O. labeo*<br>*O. labiosus*	Boxlip mullet<br>Hornlip mullet	Eastern Atlantic (Morocco to Gibraltar), Mediterranean Sea.<br>Indo-Pacific Oceans (Red Sea to the Marshall Islands, Japan, Great Barrier Reef, New Caledonia and Micronesia)
Paramugil	*P. georgii*<br>*P. parmatus*	Silver mullet<br>Broad-mouthed mullet	Western Pacific (Australia)<br>Western Pacific (Northern South China Sea to Indonesia and New Guinea)
Rhinomugil	*R. corsula*<br>*R. nasutus*	Corsula mullet<br>Shark mullet	North Indian Ocean (India, Bangladesh, Nepal and Myanmar)<br>Western Pacific (Australia and New Guinea).
Sicamugil	*S. cascasia*<br>*S. hamiltonii*	Yellowtail mullet<br>Burmese mullet	North Indian Ocean (Pakistan, India and Bangladesh).<br>North-East Indian Ocean (Myanmar)
Valamugil	*V. buchanani*<br>*V. cunnesius*<br>*V. engeli*<br>*V. formosae*<br>*V. robustus*<br>*V. speigleri*	Bluetail mullet<br>Longarm mullet<br>Kanda mullet<br>Taiwanese mullet<br>Robust mullet<br>Speigler ́s mullet	Indo-Pacific Oceans (South Africa, Philippines, Indonesia, Micronesia and Melanesia and Southern Japan)<br>Indo-West Pacific (South Africa, Somalia, Kenia, Mozambique, Madagascar and Tanzania)<br>Indo-Pacific Oceans (East Africa to the Marquesan, Tuamoto islands and the Yaeyamas)<br>North-West Pacific(Taiwan)<br>Western Indian Ocean (Inhambane, Mozambique, South Africa and Madagascar)<br>Indo-West Pacific (Pakistan, Southeast Asia, New Guinea and Chinese coasts)
Xenomugil	*X. thoburni*	Thoburn ́s mullet	Eastern Pacific (Galapagos Islands, Guatemala to Panama and Peru)

[1] Froese, R.; Pauly, D. *FishBase*. 2011 [1]; [2] Word Register of Marine Species (WoRMs) [2]; [3] Encyclopedia of Life (EOL) [3]; [4] Integrated Taxonomic Information System (ITIS) [4]; n.d. no common name defined in English.

References

1. Fishbase. Available online: http://www.fishbase.org (accessed on 25 March 2014).
2. Word Register of Marine Species, WoRMS. Available online: http://www.marinespecies.org (accessed on 25 March 2014).
3. Encyclopedia of Life, EOL. Available online: http://www.eol.org (accessed on 25 March 2014).
4. Integrated Taxonomic Information System, ITIS. Available online: http://www.ebif.gc.ca (accessed on 25 March 2014).

© 2014 by the authors; licensee MDPI, Basel, Switzerland. This article is an open access article distributed under the terms and conditions of the Creative Commons Attribution license (http://creativecommons.org/licenses/by/3.0/).
